# Scoping Review zur Identifikation und Bewertung verfügbarer digitaler Anwendungen bei bipolarer Störung

**DOI:** 10.1007/s00115-025-01857-z

**Published:** 2025-08-04

**Authors:** Taha Johanna Henning, Orestis Rakitzis, Jakob Kaminski, Linda Kokwaro, Daniel Fürstenau, Sonia Lech, Stefanie Schreiter

**Affiliations:** 1 Charité – Universitätsmedizin Berlin, corporate member of Freie Universität Berlin and Humboldt-Universität zu Berlin, Klinik für Psychiatrie und Psychotherapie, Charitéplatz 1, 10117 Berlin, Deutschland; 2 Recovery Cat GmbH, Berlin, Deutschland; 3 School of Business & Economics, Freie Universität Berlin, Berlin, Deutschland; 4 Institute of Medical Informatics, Charité – Universitätsmedizin Berlin, corporate member of Freie Universität Berlin and Humboldt-Universität zu Berlin, Berlin, Deutschland

**Keywords:** Mobile Anwendungen, Apps, Evidenz, Früherkennung, Deutschland, Mobile applications, Apps, Evidence, Early detection, Germany

## Abstract

**Hintergrund:**

Bipolare Störungen erfordern ein langfristiges Monitoring. Digitale Anwendungen, wie Smartphone-Apps, bieten Potenzial für Selbstmanagement und Früherkennung, jedoch gibt es Unsicherheiten bezüglich Verfügbarkeit, Leitlinienkonformität und Evidenz. In Deutschland fehlen spezifische digitale Gesundheitsanwendungen (DiGAs) für bipolare Störungen, was regulatorische und versorgungstechnische Herausforderungen aufzeigt.

**Ziel der Arbeit:**

Diese Übersichtsarbeit untersucht Apps für bipolare Störungen in Deutschland hinsichtlich Funktionen, Verfügbarkeit und Studienlage.

**Material und Methoden:**

Zwischen Januar und März 2025 wurde in den Datenbanken MindApps.org und MindTools.io nach Apps gesucht. Erfasst wurden Funktionen, Studienlage, Sprachen, Kostenmodelle und App-Store-Verfügbarkeit. Zudem wurden wissenschaftliche Studien zu den Apps recherchiert.

**Ergebnisse:**

Insgesamt wurden 18 Apps identifiziert, davon 11 speziell für bipolare Störungen. Die Funktionen reichen von Stimmungs- und Symptomerfassung bis zu psychoedukativen Inhalten. Sechs Apps wiesen Studien auf, jedoch ohne signifikante Verbesserungen der Symptome.

**Diskussion:**

Obwohl digitale Interventionen das Potenzial haben, die Behandlung bipolarer Störungen zu verbessern, fehlen bislang groß angelegte, methodisch robuste randomisierte kontrollierte Studien, die eine Wirksamkeit belegen. In Deutschland sind daher keine spezifischen DiGAs für diese Patient:innengruppe verfügbar. Für eine nachhaltige Verankerung in der Routineversorgung sind zudem bei einer langfristig bestehenden Erkrankung wie der bipolaren Störung verlässliche Erstattungsmodelle und umfassende Langzeitstudien erforderlich.

**Zusatzmaterial online:**

Die Online-Version dieses Beitrags (10.1007/s00115-025-01857-z) enthält eine Übersicht über die Begriffsdefinitionen sowie eine Tabelle zu Merkmalen und Funktionen digitaler Anwendungen.

## Abstract (en)

**Background:**

Bipolar disorders require long-term monitoring. Digital applications, such as smartphone apps, offer potential for self-management and early detection but there are uncertainties regarding availability, guideline conformity and evidence. In Germany, specific digital health applications (DiGAs) for bipolar disorders are lacking, highlighting regulatory and healthcare delivery challenges.

**Aim:**

This review examines apps for bipolar disorder available in Germany with respect to their functions, availability and evidence base.

**Material and methods:**

Between January and March 2025 a systematic search was conducted in the databases MindApps.org and MindTools.io for apps. Functions, research evidence, languages, cost models, and app-store availability were recorded. Additionally, scientific studies on the identified apps were reviewed.

**Results:**

A total of 18 apps were identified 11 of which are specifically for bipolar disorders. The functions range from mood and symptom tracking to psychoeducational contents. Of the apps six had published studies but none showed significant improvements in symptoms.

**Discussion:**

Although digital interventions show promise for improving the treatment of bipolar disorder, large-scale, methodologically rigorous randomized controlled trials with standardized endpoints are still lacking. In Germany no DiGAs specifically designed for this patient group are currently available. Moreover, for sustainable integration into routine care of a chronic condition such as bipolar disorder, dependable reimbursement models and comprehensive long-term studies are also required.

## Einführung

Bipolare Störungen verlaufen chronisch-episodisch mit wechselnden Phasen von Depressionen und (Hypo‑)Manien, die ein langfristiges Monitoring und Unterstützung erfordern. Digitale Anwendungen wie Smartphone-Apps bieten einen innovativen Ansatz, um Betroffene im Alltag zu unterstützen, Symptome zu erfassen, Frühwarnzeichen zu erkennen und gemeinsam mit Behandler:innen individuelle Behandlungsstrategien zu entwickeln, wie auch in Leitlinien empfohlen [1]. Weitere Anwendungsbereiche umfassen die Unterstützung von Psychotherapien durch digitale Interventionen und Feedbacksysteme sowie das Management von Medikation mit regelmäßigen Erinnerungen und Auswertungen zur Medikamenteneinnahme.

Gibt man im deutschen Apple App Store das Stichwort „bipolar“ ein, werden insgesamt 120 Apps angezeigt, die überwiegend den Kategorien Gesundheit und Fitness (53 Apps) sowie Medizin (21 Apps) zugeordnet sind. Der Fokus dieser Anwendungen liegt hauptsächlich auf psychischer Gesundheit, Therapie, Tracking und Wellness [2]. Eine Untersuchung australischer App-Stores zeigte bereits 2015 Defizite in Datenschutz und Leitlinienkonformität bei Apps zur Anwendung für bipolare Störungen [2]. Neuere Analysen zeigen, dass nur 56 % der Apps explizit die bipolare Störung adressieren und kaum Wirksamkeitsnachweise existieren [3]. Insgesamt warnten die Autor:innen vor einer einfachen App-Suche und fanden auch schädliche und irreführende Anwendungen.

Die Evidenz zur Effektivität digitaler Anwendungen bei bipolaren Störungen ist begrenzt. Eine Metaanalyse (2019/2020) mit 10 Studien (darunter 7 randomisierte kontrollierte Studien [RCTs]) fand kleine, aber signifikante Effekte: Smartphone-Interventionen, die psychoedukative Inhalte oder andere therapeutische Maßnahmen zur aktiven Symptomreduktion beinhalten, reduzierten sowohl manische (Hedges’ g −0,19) als auch depressive Symptome (g −0,28). Monitoringsysteme, die sich ausschließlich auf die Erfassung und Überwachung von Symptomen konzentrieren, zeigten hingegen nur eine signifikante Wirkung auf manische Symptome [4]. Besonders wirksam waren Apps mit Psychoedukation oder regelmäßigen In-App-Kontakten mit Therapeut:innen, wie z. B. durch Onlinegespräche oder Messaging. Eine Empfehlung zur Integration in die bestehende psychotherapeutische Behandlung vor Ort wurde in den untersuchten Studien nicht explizit untersucht. Reine Selbstbeobachtung reichte meist nicht aus, und die Effektstärken blieben insgesamt gering.

Eine systematische Übersichtsarbeit aus dem Jahr 2022 kommt zu einem kritischeren Fazit [5]. Die Metaanalyse fand keinen überzeugenden Nachweis, dass Smartphone-basierte Interventionen die Intensität depressiver oder manischer Symptome signifikant verringern oder die Lebensqualität verbessern. Keiner der 6 RCTs zeigte eine Wirksamkeit bei der Reduktion der Symptome oder Verbesserung der Funktionsfähigkeit im Vergleich zu aktiven Kontrollgruppen (z. B. Placebo-Apps ohne therapeutischen Inhalt). Die Wirksamkeit zeigte sich nur im Vergleich zu inaktiven Kontrollbedingungen wie „treatment as usual“ (TAU). Die Autor:innen führen diese Diskrepanz auf die hohe Heterogenität der Studien (App-Typen, Zielgruppen, Methodik) sowie auf das Fehlen großer, langfristiger RCTs mit robusten Endpunkten zurück. Insgesamt ist die Evidenzlage noch uneinheitlich: Einige Studien zeigen positive Effekte auf die Stimmungsstabilität, jedoch fehlt ein definitiver Wirksamkeitsnachweis im Vergleich zu Standardbehandlungen.

Neben aktivem Tracking (Stimmungs- und Symptomerfassung) gibt es zunehmend Ansätze des passiven Trackings („digital phenotyping“), bei denen Smartphones oder Wearables kontinuierlich Daten zu Aktivität, Schlaf und sozialer Interaktion erfassen. Aktuelle Übersichtsarbeiten [6] zeigen erste Hinweise auf die Machbarkeit, berichten jedoch auch von uneinheitlichen Ergebnissen und hoher Heterogenität der Studien. Es fehlen einheitliche, groß angelegte, longitudinale Untersuchungen und standardisierte Leitlinien für den Einsatz in der Versorgung.

In Deutschland ermöglichen die digitalen Gesundheitsanwendungen (DiGAs) – bekannt als „Apps auf Rezept“ – die Einführung wirksamer und sicherer digitaler Tools in die Regelversorgung. Derzeit konzentrieren sich die verfügbaren DiGAs hauptsächlich auf die Behandlung von Depressionen und Angststörungen [7]. Spezifische DiGAs für bipolare Störungen fehlen bislang aufgrund regulatorischer und marktbezogener Hürden. Dieses Defizit führt zu ungleichem Zugang zu innovativen therapeutischen Ansätzen und verstärkt bestehende soziale und gesundheitliche Ungleichheiten, da Gruppen, die ohnehin eingeschränkten Zugang zu Ressourcen wie Psychotherapie haben, von diesen Technologien ausgeschlossen bleiben [8]. Das Fehlen von DiGAs für bipolare Störungen zeigt den Bedarf an weiterer Forschung und Entwicklung bipolar spezifischer digitaler Tools. Dieser Artikel bietet einen Überblick über digitale Apps für bipolare Störungen, die auf Plattformen für psychische Gesundheitsangebote (z. B. App-Stores) in Deutschland verfügbar sind, und fasst dazu Studienergebnisse, Funktionen, Spezifität sowie ihre Nutzbarkeit in Deutschland zusammen. Entwicklungspotenziale und Barrieren sowie die Frage nach notwendigen Schritten, um digitale Anwendungen ins deutsche Versorgungssystem zu implementieren werden diskutiert.

## Methoden

Zwischen Januar und März 2025 wurde ein Scoping Review zu mobilen Anwendungen für bipolare Störungen durchgeführt. Ziel war es, in den gängigen App-Stores verfügbare Apps systematisch zu identifizieren und ihre Funktionen sowie unterstützende Evidenz zu kategorisieren. Die Suche erfolgte in den wissenschaftlich kuratierten Datenbanken mindapps.org^1^ und MindTools.io^2^. Zusätzlich wurden Mindpax (Mindpax s.r.o., Prag, Tschechien) und MONARCA (Monsenso A/S, Kopenhagen, Dänemark) aufgrund ihrer Erwähnung in relevanten Publikationen eingeschlossen. Die Suchbegriffe umfassten „bipolar“ und „mood“, um eine breite Abdeckung relevanter Anwendungen zu gewährleisten.

Einschlusskriterien waren (I) die explizite Adressierung der bipolaren Störung bzw. Anwendungen, die die Stimmung („mood“) als Zielsymptom unterstützen, (II) zudem wurden ausschließlich Apps berücksichtigt, die in deutschen App-Stores auffindbar sind und zum Download zur Verfügung stehen. Die Datenextraktion erfolgte anhand von Beschreibungen auf den Plattformen mindtools.io und mindapps.org sowie aus den App-Beschreibungen im Apple (Apple Inc., Cupertino, Kalifornien, USA) und Google Play Store (Google LLC, Mountain View, Kalifornien, USA) bzw. der publizierten Studien. Die zentralen Merkmale wie Funktionen, Studienlage, Sprachen, Kostenmodelle sowie die Verfügbarkeit wurden extrahiert. Die Anwendungen wurden durch zwei Autor:innen bewertet. Ergänzend dazu wurde zu den eingeschlossenen Anwendungen eine Literaturrecherche in PubMed und Google Scholar durchgeführt, um Studien zu den identifizierten Apps zu finden.

## Ergebnisse

Insgesamt wurden 230 digitale Anwendungen gescreent, von denen 18 die Einschlusskriterien erfüllten. Eine detaillierte Darstellung der einzelnen Apps, einschließlich ihrer Funktionen, Studienlage und Merkmale, ist in Tab. 1 im Zusatzmaterial online zu finden. Die Analyse ergab dabei folgende Kernergebnisse:

### Spezifität

Elf der identifizierten Apps wurden speziell für bipolare Störungen entwickelt, während 7 Anwendungen Funktionen anbieten, die auf das Zielsymptom Stimmung ausgerichtet sind, jedoch nicht ausschließlich auf die bipolare Störung.

### Funktionen

Alle Apps bieten ein aktives Symptom- und Stimmungstracking an. Zwei der Anwendungen gaben an, den Mood Disorder Questionnaire (MDQ; [9]) als standardisiertes Instrument zu nutzen, während eine weitere Anwendung den Patient Health Questionnaire 9 (PHQ‑9; [10]) und Generalizied Anxiety Disorder Scale 7 (GAD‑7; [11]) zur Erfassung von Depressions- und Angstsymptomen einsetze. Weiterhin erfasst das aktive Tracking je nach Anwendung neben der Stimmung auch Aspekte wie Schlaf, Medikationseinnahme, Ernährung, Ressourcen oder Aktivitäten. Weitere häufige Funktionen umfassen das Führen eines Journals, Berichts- und Analysefunktionen sowie psychoedukative Inhalte. Vier der Anwendungen („Mindpax“, „MONARCA“, „Effecto Symptom & Mood Tracker“ (Medical Score GmbH, Panevezys, Litauen) und „Symptom & Mood Tracker“ (BEARABLE Limited, Biggin Hill, Vereinigtes Königreich)) gaben an, passiv Schlaf, physische Aktivität, soziale Aktivität, Mobilität, Smartphonenutzung sowie Daten über die Synchronisation mit Drittanbietern wie Google Fit oder Apple Health zu nutzen. Zudem enthalten 11 Apps Module zur Routineplanung und Erinnerungsfunktionen. Alle Anwendungen geben an, über eine Datenschutzrichtlinie zu verfügen.

### Sprachen, Kosten und Verfügbarkeit

Sieben der Apps sind auf Deutsch verfügbar („eMoods Bipolar Mood Tracker“ (Yottaram LLC, Buxton, Maine, USA), „Mood, BPD, Bipolar Feeltracker“ (Custom Arts Ltd, Hove, Vereinigtes Königreich), „Bipolar Mood Tracker“ (Adam Cziko, Wien, Österreich), „Daylio“ (Relaxio s.r.o, Bratislava, Slowakei), „Mindpax“, „MONARCA“ und „Mood Tracker Journal“ (Reflexio Team, Huelva, Spanien)), während alle 18 Anwendungen in englischer Sprache angeboten werden. Der Download der Apps erfolgt in den meisten Fällen zunächst kostenfrei, wobei 14 Anwendungen kostenpflichtige In-App-Käufe oder Abonnements anbieten. Bezüglich der Plattformen sind die meisten Anwendungen sowohl im Apple App Store als auch im Google Play Store erhältlich, wenngleich 7 Apps ausschließlich für ein Betriebssystem verfügbar sind. Die beiden Apps Mindpax.me und MONARCA stehen zwar zum Download zur Verfügung, eine Nutzung war jedoch aufgrund fehlgeschlagener Registrierungsversuche zweier Autor:innen nicht möglich.

### Evidenzlage

Nur 6 Apps wurden in Studien untersucht (darunter 3 RCTs, ein Fallbericht, 2 Usability-Testungen und eine Beobachtungsstudie). Im Folgenden werden die Studienergebnisse zu diesen Apps zusammengefasst, um einen Überblick über ihre Wirksamkeit und Nutzbarkeit zu geben.

#### KIOS und eMoods

In einer 52-Wochen-RCT (*n* = ca. 130) zeigte die Selbstmonitoring-App mit Feedback KIOS (Biomedical Development Corporation, San Antonio, Texas, USA) zwar eine signifikant höhere Adhärenz in der App-Nutzung (88 % vs. 74 %, *p* = 0,03) und Zufriedenheit im Vergleich zur reinen Mood-Tracking-App ohne Feedback (eMoods), jedoch keine signifikante Reduktion depressiver oder manischer Symptome [2]. Eine begleitende Pilotstudie über 2 Monate bestätigte die hohe tägliche Nutzung [12].

#### Daylio

Daylio (nicht bipolarspezifisch) wurde in 2 Case-Reports und einer Usability-Testung mit 14 gesunden Personen beschrieben (einfache Bedienung, gute technische Zuverlässigkeit, jedoch Probleme beim Sign-in; [13–15]). RCTs zur Wirksamkeit fehlen. Eine Panel-basierte Studie von Baumel et al. zeigte oft geringe langfristige Adhärenz, auch bei Daylio [16].

#### Mindpax

Das Mindpax-System aus Tschechien kombiniert passive Datenerfassung durch Wearable (Aktivitäts- und Schlafparameter) mit wöchentlichen Stimmungsbewertungen (dafür entwickelter Aktibipo Self-rating Questionnaire [ASERT]; [17]). Eine 2‑jährige Beobachtungsstudie (99 Personen mit bipolarer Störung) zeigte signifikante Korrelationen zwischen App-Selbstberichten und klinischen Skalen (*p* < 0,001) sowie Potenzial zur Frühwarnung von Episoden [17]. Allerdings liegen bisher keine RCTs vor, die einen signifikanten Effekt auf die Symptomatik oder funktionelle Outcomes belegen; die Adhärenz lag in der 2‑jährigen Kohortenstudie bei 78 % [17]. Eine weitere 12-monatige Beobachtungsstudie ist registriert [18].

#### MoodMission (MoodMission Pty Ltd, Brunswick, Victoria, Australien) 

Diese allgemeine Stimmungs-App mit KVT(kognitive Verhaltenstherapie)-Elementen wurde in einer RCT an einer gemischten Community-Stichprobe untersucht (im Vergleich mit anderen Mood-Tracking/KVT-Apps MoodKit (Thriveport, Los Angeles, Kalifornien, USA) und MoodPrism (MoodPrism Pty Ltd, Warrandyte, Victoria, Australien) gegen Warteliste, 30 Tage, *N* = 226; [19]). Im Vergleich zur Warteliste zeigten die Apps MoodKit und MoodMission signifikante Reduktionen depressiver Symptome. Zudem zeigte MoodMission eine Usability-Testung mit positiven Ergebnissen [19].

#### MONARCA

Das MONARCA(MONitoring, treAtment and pRediCtion of bipolAr disorder)-System (von Monsenso) ist ein früh entwickeltes Smartphone-Monitoring-Tool mit einem Feedback-Loop zu Behandelnden. In einem 6‑monatigen RCT (*n* = 78 Personen mit bipolarer Störung; [20]) zeigte es trotz guter Akzeptanz und täglicher Nutzung keine signifikante Reduktion von Symptomen im Vergleich zur Standardbehandlung, auch keine Verbesserungen in Lebensqualität oder funktionellem Status [20]. Auch eine weitere RCT (*n* = 129) zeigte keine signifikante Symptomreduktion [12]. Derzeit wird eine RCT mit einem angepassten MONARCA-System durchgeführt, das Monitoring mit und ohne Feedback-Loop zu Behandelnden vergleicht [21].

#### Moodfit

Moodfit (Roble Ridge Software LLC, Palo Alto, Kalifornien, USA) wurde in einer Expert:innen-basierten Bewertungsstudie untersucht, in der 17 Kliniker:innen 3 Apps hinsichtlich Benutzerfreundlichkeit und klinische Nützlichkeit für Depressionen nach traumatischen Hirnverletzungen bewerteten [22]. Moodfit erhielt eine durchschnittliche Bewertung von 4,28 von 5 Punkten, wobei Funktionen wie psychoedukative Inhalte und Symptommanagement nur nach der kostenpflichtigen Testphase verfügbar waren [22].

## Diskussion

Unsere Übersichtsarbeit zeigt ein breites internationales Angebot an Apps für bipolare Störungen, von denen einige auch in deutscher Sprache verfügbar sind. Jedoch liegen nur wenige Anwendungen mit robusten Studien vor, welche wiederum nur unzureichende Wirksamkeitsnachweise erbringen. Gleichzeitig werden neue Anwendungen häufig speziell für Studien entwickelt, schaffen es aber nicht bis zur Marktreife. Dies führt zu einem doppelten Dilemma: einerseits frei verfügbare Anwendungen ohne Evidenz, andererseits Studien ohne ein öffentlich zugängliches Produkt.

Bei einem Großteil der verfügbaren Apps bleibt auf Basis der Publikationen unklar, inwieweit diese evidenzbasiert entwickelt wurden und entsprechenden Leitlinien für bipolare Störungen folgen. Von den 6 Anwendungen mit publizierten Studien sind nur 4 (Moodfit, MoodMission, eMoods, Daylio) in Deutschland nutzbar. Zudem ist MoodMission nach 24 h kostenpflichtig (und nicht erstattungsfähig). Die Ergebnisse bestätigen frühere Reviews englischsprachiger Apps, die geringe Evidenz und Leitlinienkonformität zeigten [2, 3]. Die vorliegende Evidenz ist methodisch heterogen und meist explorativ. Kleine Stichproben, kurze Beobachtungszeiträume und unterschiedliche Kontrollbedingungen erschweren direkte Vergleiche. Für die 3 Anwendungen mit RCTs (eMoods, MoodMission, Monsenso) zeigten sich keine signifikanten Effekte auf Symptomatik oder Funktionsniveau bei bipolaren Störungen. Die einzige App mit positiven Effekten auf depressive Symptome (MoodMission) wurde in einem Community-Sample untersucht, nicht unter Personen mit klinisch relevanten Störungen [19]. Diese Befunde bestätigen Metaanalysen, die keine oder nur geringe Effekte auf Symptomreduktion fanden [4]. Insbesondere bei bipolarer Störung scheinen alternative Endpunkte zur reinen Symptomreduktion und längere Beobachtungszeiträume essenziell, da sich die Häufigkeit von Episoden individuell stark unterscheidet und die Aussagekraft kurzer Messperioden durch phasenabhängige Schwankungen begrenzt wird.

Die meisten Apps setzen auf aktives Tracking und Journaling (z. B. eMoods, Bipolar UK Mood Tracker (Bipolar UK, London, Vereinigtes Königreich)), einige integrieren Psychoedukation (MONARCA, Flamingo – Mood Tracker Diary (Davin App LTD, London, Vereinigtes Königreich)) oder KVT-Elemente (MoodMission). Eine Metaanalyse zeigt, dass Anwendungen mit therapeutischen oder psychoedukativen Inhalten reinen Monitoringanwendungen überlegen sind [4]. Passives Tracking (Aktivität, Schlaf, Kommunikation) liefert erste Hinweise auf eine Früherkennung von Rückfällen und Phasenwechseln. Vier Apps nutzen diese Daten explizit. Die Beobachtungsstudie zu Mindpax (mit Wearable) ergab vielversprechende Vorhersagewerte für Krankheitsepisoden (z. B. Manie: Sensitivität 64,9 %, Spezifität 89,9 %), jedoch war die App nicht praktisch auf dem freien Markt nutzbar [17]. Für eine klinische Integration sind größere, methodisch robuste RCTs erforderlich, die Lebensqualität und Alltagsfunktion erfassen. Zudem müssen Datenschutz und regulatorische Vorgaben erfüllt werden. Das DiGA-Konzept fokussiert derzeit vorwiegend auf Anwendung für Depression oder Angststörungen, nicht für bipolare Störungen oder psychotische Erkrankungen. Dies unterstreicht den Bedarf, internationale Entwicklungen zu adaptieren und bipolarspezifische digitale Lösungen – unter strengen Qualitäts- und Datenschutzvorgaben – in den deutschen Markt zu überführen und auch entsprechende Erstattungsmodelle zu entwickeln [23].

Basierend auf Metaanalysen, die darauf hinweisen, dass die langfristige Wirksamkeit und der Nutzen digitaler Tools in der Routineversorgung noch nicht ausreichend belegt sind, sollten diese zunächst nur als ergänzende Maßnahmen zur herkömmlichen Therapie betrachtet werden. Gleichzeitig kann es hilfreich sein, wenn diese Anwendungen Behandler:innen bekannt sind, da sie Patient:innen frei zugänglich sind. Ohnehin zeigten Metaanalysen, dass Apps mit Behandelndenintegration überlegen sind [4]. Die MONARCA-Studien deutet jedoch darauf hin, dass reines Tracking – selbst mit ärztlicher Einbindung – nicht ausreicht, um Symptome signifikant zu verbessern [12, 20]. Eine laufende RCT (SmartBipolar Trial) testet derzeit App-Monitoring mit und ohne therapeutischen Feedback-Loop [21]. Wirksam scheinen digitale Anwendungen vor allem bei multimodalem Einsatz – etwa zur Phasenfrüherkennung, Therapieunterstützung und Medikamentencompliance. Recovery Cat (Recovery Cat GmbH, Berlin, Deutschland) [8, 24], ein in Deutschland entwickeltes, integriertes System, das individualisierte Therapieplanung, Medikationserinnerungen und personalisiertes Monitoring integriert und sich explizit auch an Personen mit bipolarer Störung richtet, verfolgt diesen Ansatz. Die Anwendung ist jedoch bisher nur per Selektivvertrag mit einer Krankenkasse in wenigen Kliniken zugänglich, auch fehlt bisher ein Wirksamkeitsnachweis mittels RCT.

Internationale Studien zeigen eine hohe initiale Akzeptanz digitaler Tools, wobei die langfristige Nutzung oft durch mangelnde Adhärenz begrenzt wird, die häufig sehr heterogen oder gar nicht erfasst wird [25]. Dies dürfte auch im deutschen Versorgungssystem eine zentrale Herausforderung darstellen. Obwohl Mental-Health-Apps von den Nutzer:innen allgemein positiv wahrgenommen werden, müssen Faktoren wie Benutzerfreundlichkeit, inhaltlicher Nutzen und Datenschutz berücksichtigt werden, um die langfristige Akzeptanz zu maximieren [26]. In einer aktuellen Studie (DigiNavi) wird derzeit untersucht, inwieweit speziell im Hinblick auf verfügbare Apps geschulte Mitarbeiter:innen (sog. „digitale Navigator:innen“) das Engagement und die Adhärenz bei der Nutzung digitaler Gesundheitsanwendungen für psychische Erkrankungen verbessern können [27, 28]. Ein Ansatz zur Verbesserung der Nutzerbindung könnte die Integration in ein Blended-Care-Modell sein, was auch im deutschen Konzept DiGA möglich ist. Blended Care bezeichnet dabei die verzahnte Versorgung, in der digitale Anwendungen gezielt mit persönlichen Kontakten kombiniert werden, um Vorteile beider Formate zu nutzen und eine kontinuierliche Betreuung sicherzustellen.

Apps können das Empowerment und Selbstmanagement fördern, indem sie Nutzer:innen helfen, Zusammenhänge zwischen Auslösern (z. B. Stress, Schlafentzug) und Stimmung zu erkennen [29]. Dadurch steigt die Patient:innenautonomie und -compliance. Allerdings kann engmaschige Selbstbeobachtung auch belastend wirken: Tägliches Protokollieren kann Stress oder Ängste verstärken und zur Überinterpretation normaler Stimmungsschwankungen führen, insbesondere wenn keine Bewältigungsstrategien vermittelt werden, was bei bisher papierbasiertem Monitoring wenig Berücksichtigung fand. Ebenso kann dieser Überfokus fördern, dass Betroffene ihre Gefühle als potenziell gefährlich wahrnehmen, was zu Hypervigilanz und Selbststigmatisierung führen kann [30]. Onlinebasierte Tagebücher ermöglichen eine intuitive Dateneingabe, automatische Analysen und die Verknüpfung mit Gesundheitsdaten. Zudem erleichtern sie die Kommunikation mit Therapeut:innen, indem Trends übersichtlich dargestellt und sicher geteilt werden. Die Nutzung von Apps in der Versorgung erfordert jedoch neue Arbeitsabläufe: Wer wertet die Daten aus und reagiert darauf? Viele Patient:innen nutzen bereits frei verfügbare Anwendungen, doch Praxen und Kliniken sind oft nicht darauf eingerichtet, kontinuierlich Patient:innendaten zu monitorieren. Ohne klare Verantwortlichkeiten können Stimmungsverläufe im Praxisalltag untergehen. Zudem droht eine Datenflut: Ohne automatisierte Vorselektion (z. B. Alarm bei kritischen Veränderungen) kann das Personal überfordert sein. Auch das Erklären und Nachhalten der App-Nutzung erfordert zusätzliche Zeit – ein Aufwand, der im engen Versorgungstakt schwer abzubilden ist, zumal eine Vergütung fehlt.

Diese Übersichtsarbeit weist mehrere Limitationen auf. Erstens könnten durch die Fokussierung auf zwei Datenbanken (MindApps.org, MindTools.io) relevante Anwendungen oder Studien fehlen. Zweitens erschweren methodische Heterogenität, kleine Stichproben und kurze Beobachtungszeiträume die Vergleichbarkeit. Drittens wurden die Apps nicht systematisch getestet, und Datenschutz- sowie Zulassungsinformationen waren teils unzureichend. Es erfolgte keine Registrierung des Reviews, da es sich nicht auf wissenschaftliche Datenbaken primär fokussierte.

Für die Implementierung in das deutsche Gesundheitssystem sollten zukünftig die Konformität mit Datenschutzrichtlinien und Fragen von Datensicherheit (bspw. Serverstandorte) genauer überprüft und standardisiert und verständlich für Nutzende dokumentiert werden. Darüber hinaus sollte untersucht werden, inwieweit digitale Anwendungen mit bestehenden klinischen Leitlinien übereinstimmen und welche Maßnahmen erforderlich sind, um eine bessere Leitlinientreue sicherzustellen.

## Fazit für die Praxis

- Trotz zahlreicher Apps mit Funktionen wie Tracking, Psychoedukation und Wearable-Integration für Personen mit bipolaren Störungen in deutschen App-Stores besteht keine ausreichende Evidenz für deren Wirksamkeit. Darüber hinaus besteht ein Defizit hinsichtlich der Transparenz über eine evidenzbasierte Entwicklung der frei verfügbaren Apps. Dagegen sind Anwendungen, die evidenzbasiert entwickelt wurden, nicht ohne Hindernisse zugänglich.
- Zugleich zeigen auch systematische Übersichtsarbeiten, die international verfügbare Anwendungen einbeziehen, bisher keine ausreichenden Wirksamkeitsnachweise gegenüber Standardbehandlung bei der bipolaren Störung, auch wenn es Hinweise gibt, dass Anwendungen mit Selbstmanagement, Psychoedukation und therapeutischer Rückkopplung Vorteile gegenüber reinen Monitoring- oder Selbstmanagementanwendungen zeigen.
- Die fehlende Entwicklung digitaler Gesundheitsanwendungen (DiGAs) im deutschen Raum für Menschen mit schweren psychischen Erkrankungen wie bipolaren Störungen stellt einen erheblichen Versorgungsmangel dar, bei gleichzeitig hohen Einbußen in Lebensqualität und Funktionsniveau.

## Supplementary Information

Infobox: Wichtige Begriffe in der digitalen Gesundheitsversorgung im Bereich Psychiatrie und Psychotherapie

Tabelle 1 Merkmale und Funktionen digitaler Anwendungen

## Footnotes

1 Die Website mindapps.org (mHealth Index and Navigation Database [MIND]) ist eine interaktive, kostenfreie Datenbank für die gezielte Suche nach mentalen Gesundheits-Apps. Sie wird von Expert:innen der Harvard Medical School betrieben und nutzt einen standardisierten Bewertungsrahmen mit 105 Kriterien (z. B. Datenschutz, Evidenz, Benutzerengagement). Die Plattform wird regelmäßig aktualisiert (alle 100–180 Tage).

2 Die Website MindTools.io ist eine interaktive, kostenfreie Plattform für digitale Anwendungen zur Förderung von Wohlbefinden und mentaler Gesundheit. Sie verwendet das Enlight-Rating-Schema zur Bewertung von Qualität, Usability und therapeutischer Überzeugungskraft, basierend auf kontinuierlicher Evaluierung durch geschulte Expert:innen.
