# Supplementary material for: Scoping review on the identification and evaluation of available digital applications for bipolar disorder
Source: Nervenarzt. 2025 Aug 4;96(5):423–31. [Article in German] doi: 10.1007/s00115-025-01857-z (PMC12411593; doi:10.1007/s00115-025-01857-z)
Supplement: Supplementary file 2 — Tabelle 1 Merkmale und Funktionen digitaler Anwendungen [file 115_2025_1857_MOESM2_ESM.pdf]

Tabelle 1 **Merkmale und Funktionen digitaler Anwendungen**

Name (Entwickler)	Monitoring* (Aktiv und passiv)	Journal/ Notizbuch	Berichte und Analysen	Psychoedukation	Routine- und Zielplanung	Zusätzliche Funktionen	Sprache	Kosten	Qualität aus Nutzersicht**	Download	Studien
Bipolar Test (Inquiry Health LLC)	*Aktiv:* Symptome (MDQ)		Erklärung der MDQ-Testergebnisse	x		Kontaktmöglichkeiten zu Coach oder Therapeut	englisch	kostenfrei	-	Android, iOS	-
eMoods Bipolar Mood Tracker (eMoods)	Stimmung, Symptome, Schlaf und Medikation	x	Erstellung von Berichten und grafischen Darstellungen			Datensicherheit lokal	7 Sprachen u.a. deutsch, englisch, russisch	kostenfreier Download, In-App-Abonnement: 1,99 € / Monat, 11,49 € / Jahr	2,9 von 5 (schlecht)	Android, iOS	Vergleich von KIOS und eMoods in 52-Wochen-RCT: keine sign. Symptomreduktion [25].
Bipolar Test (Baris Sarer)	*Aktiv:* Stimmung und Symptome (MDQ)		Teilen von Testergebnissen mit medizinischen Fachkräften	x			englisch	kostenfrei	-	iOS	-
Breeze: Start Self-Discovery (Basenji Apps)	*Aktiv:* Stimmung und Symptome	x	Statistiken zur Selbstreflexion und Fortschritten	x	Individualisierte Routinepläne und Zielsetzung	Austausch in einer Community, Bildgalerie/Hoffnungs-Board	englisch	kostenfreier Download, In-App-Abonnement: 7-Tage-Testversion für $1, danach $29,99 / Monat	-	Android, iOS	-
Bipolar UK Mood Tracker (BipolarUK)	*Aktiv:* Stimmung, Emotionen und Medikation	x	Übersicht und Berichte für medizinische Fachpersonen			Kontaktmöglichkeiten zum Gesundheitsdienstleister und Peersupport	englisch	kostenfrei	-	Android, iOS	-
Mood, BPD, Bipolar·Feeltracker (Custom Arts)	*Aktiv:* Stimmung und Gefühle	x	Analyse der Daten, erstellen von Berichten (PDF, CSV, Excel und JSON)		Erinnerungsfunktion	Face ID/ Touch ID für Datenschutz und Sicherheit	12 Sprachen u.a. deutsch, englisch, türkisch	kostenfreier Download, In-App-Abonnement: Werbung entfernen 2,99€/ Monat, 22,99€ /Jahr	-	iOS	-
Bipolar Mood Tracker° (Adam Cziko)	Stimmung, Medikation, Symptome, Schlaf und körperlicher Gesundheit	x	Erstellung von PDF- oder CSV-Berichten zur Weitergabe an Ärzt*innen		Erinnerungsfunktion	Journalschutz mit Face ID oder Touch ID	7 Sprachen u.a. deutsch, englisch, russisch	kostenfreier Download, In-App-Abonnement	-	iOS	-
Flamingo - Mood Tracker, Diary (Cumali Han UNLU)	*Aktiv:* Stimmung	x	Stimmungsdiagramme, Erstellung von PDF- oder CSV-Berichten	Psychoedukation zu Technik aus der positiven Psychologie		Face ID/ Touch ID für Datenschutz und Sicherheit	englisch	kostenfreier Download, In-App-Abonnement: Flamingo Pro - $3.49/Woche	-	iOS	-
Daylio (Habitics/ Relaxio s.r.o.)	*Aktiv:* Stimmung und Aktivitäten	x	Stimmungsverläufe, CSV-Download (kostenpflichtig)		Erinnerungsfunktion und Zielsetzung	Journaling mit Sprachmemos und Fotos	24 Sprachen u.a. deutsch, englisch, türkisch	kostenfreier Download, In-App-Abonnement nach einer Woche: 4,99€/Monat, 35,99€/ Jahr	4 von 5 (gut)	Android, iOS	Case Reports: einfache Bedienung und technische Zuverlässigkeit jedoch Probleme bei Sign-In [9,10,17], geringe langfristige Adhärenz [6]
*Mindpax.me (MINDPAX)*²*	*Aktiv:* Stimmung, Medikation *Passiv:* Schlaf, Schritte, Kalorien		Analyse von Stimmung, Medikation und Aktivitäten				deutsch, englisch, tschechisch	kostenfreier Download, In-App-Abonnement nach einer Woche: 22,01€/	-	Android, iOS	2-jährige Kohortenstudie: sign. Zusammenhang zw. App-Selbstberichten und klinischen Skalen [4]

								Monat, 220,12€ /Jahr			
*MONARCA (Monsenso)*²*	*Aktiv:* Stimmung, Medikation, Schlaf, Aktivitätsniveau *Passiv:* physische Aktivität, soziale Aktivität, Mobilität und Smartphonenutzung	x	Übersicht und Berichte für medizinische Fachpersonen	x		Warnungen basierend auf erkannten Mustern, die auf eine Verschlechterung hinweisen könnten	13 Sprachen u.a. deutsch, englisch, dänisch	kostenfreier Download, In-App-Abonnement nach einer Woche	-	Android, iOS (Monsenso)	6-monatige RCT: keine sign. Reduktion von Symptomen, Lebensqualität oder funktionellem Status [13,14]
Effecto Symptom & Mood Tracker (Health Score/ Medical Score)	*Aktiv:* Stimmung, Symptome, Medikation, Aktivitäten *Passiv:* Schlaf, physische Aktivität durch Synchronisation mit Google Fit oder Apple Health	x	Datensammlung und Auswertung	x	Zielsetzung und Gewohnheitsbildung		englisch	kostenfreier Download, In-App-Abonnement, 29,99 $ alle 3 Monate, 59,99$/ Jahr	-	Android, iOS	-
Symptom & Mood Tracker (Bearable)	*Aktiv:* Stimmung, Medikation, Symptome, Aktivitäten *Passiv:* Schlaf, physische Aktivität durch Synchronisation mit Google Fit oder Apple Health	x	Analyse von Symptomen und Aktivitäten, Berichterstellung		Erinnerungsfunktion und Zielsetzung		englisch	kostenfreier Download, Premium In-App-Abonnement: 5,99 €–89,99 €	-	Android, iOS	-
Mood Tracker Journal (Reflexio Team)	*Aktiv:* Stimmung und Symptome	x	Stimmungsverläufe		Erinnerungsfunktion und Zielsetzung		deutsch, englisch	kostenfreier Download, In-App-Kauf: 2,59 - 4,99 €	-	Android	-
MoodMission - Cope with Stress (MoodMission Pty Ltd)	*Aktiv*: Stimmung			Psychoedukation zur Unterstützung des Wohlbefindens	Zielsetzung	Entspannungsübungen, körperliche Aktivitäten und Coping-Statements	englisch	kostenfreier Download, In-App-Abonnement nach 24 Stunden notwendig	-	Android, iOS	RCT an gemischter Community-Stichprobe: sign. Reduktion depressiver Symptome und positive Usability-Testung [5]
Mood Log (AR Productions Inc.)	*Aktiv:* Stimmung, Medikation und Symptomen	x	Analyse von Stimmungsverläufen, Ereignissen und Symptomen, Datenexport		Erinnerungsfunktion		englisch	kostenfreier Download, In-App-Kauf: 2,99 €	-	Android	-
iMoodJournal (Inexika Inc.)	*Aktiv:* Stimmung und Symptome	x	Analyse von Stimmungs- und Aktivitätsmustern		Erinnerungsfunktion	Journaling mit Fotos	4 Sprachen u.a. englisch, französisch, russisch	2,99 €	3,3 von 5 (angemessen)	iOS	-
Moodfit (Roble Ridge Software LLC)	Stimmung, Medikation, Symptome, Schlaf, Interaktionen, Ernährungsverhalten	x	Analyse von Stimmungsmustern und Verhaltensweisen	Kognitive Umstrukturierung, Achtsamkeits- und Atemübungen	Erinnerungsfunktion und Zielsetzung	Selbstbewertungstests für Depression (PHQ-9) und Angst (GAD-7) Gemeinschaftsraum mit Live-Meditationen und Austausch	englisch	kostenfreier Download, Premium In-App-Abonnement	5 (sehr gut)	Android, iOS	Expertenbasierten Bewertungsstudie: durchschnittliche Benutzerfreundlichkeit und starke Funktionseinschränkung nach kostenloser Testphase [29]

*Aktives und passives Tracking angegeben, wenn in Quelle explizit erwähnt; **Nutzerbewertung von mindtools.io; MDQ = Mood Disorder Questionnaire; RCT = randomisierte kontrollierte Studie; PHQ-9 = Patient Health Questionnaire-9; GAD-7 = Generalized Anxiety Disorder-7 item scale.
